# Effects of Interleukin-4 or Interleukin-10 gene therapy on trinitrobenzenesulfonic acid-induced murine colitis

**DOI:** 10.1186/1471-230X-13-165

**Published:** 2013-12-06

**Authors:** Jing Xiong, Ying-Hao Lin, Li-Hong Bi, Ji-De Wang, Yang Bai, Si-De Liu

**Affiliations:** 1 Guangdong Provincial Key Laboratory of Gastroenterology, Department of Gastroenterology, Nanfang Hospital, Southern Medical University, Guangzhou 510515, China

**Keywords:** Inflammatory bowel disease, Interleukin-4, Interleukin-10, TNBS-induced colitis, Gene therapy

## Abstract

**Background:**

Inflammatory bowel disease (IBD) is characterized by disturbance of pro-inflammatory cytokines and anti-inflammatory cytokines. Previous studies have demonstrated the effect of anti-inflammatory cytokines, such as interleukin-10 (IL-10) or IL-4 on IBD, but their data were controversial. This study further investigated the effect of IL-4 (IL-4), IL-10 and their combination on treatment of trinitrobenzenesulfonic acid (TNBS)-induced murine colitis.

**Methods:**

pcDNA3.0 carrying murine IL-4 or IL-10 cDNA was encapsulated with LipofectAMINE 2000 and intraperitoneally injected into mice with TNBS-induced colitis. The levels of intestinal IL-4 and IL-10 mRNA were confirmed by quantitative-RT-PCR. Inflamed tissues were assessed by histology and expression of interferon (IFN)-γ, tumor necrosis factor (TNF)-α and IL-6.

**Results:**

The data confirmed that IL-4 or IL-10 over-expression was successfully induced in murine colon tissues after intraperitoneal injection. Injections of IL-4 or IL-10 significantly inhibited TNBS-induced colon tissue damage, disease activity index (DAI) and body weight loss compared to the control mice. Furthermore, expression of IFN-γ, TNF-α and IL-6 was markedly blocked by injections of IL-4 or IL-10 plasmid. However, there was less therapeutic effect in mice injected with the combination of IL-4 and IL-10.

**Conclusions:**

These data suggest that intraperitoneal injection of IL-4 or IL-10 plasmid was a potential strategy in control of TNBS-induced murine colitis, but their combination had less effect.

## Background

Inflammatory bowel disease (IBD), including Crohn’s disease (CD) and ulcerative colitis (UC), is characterized by chronic and relapsing inflammation of the gastrointestinal tract that ultimately leads to the destruction of the intestinal tissues. Epidemiologically, IBD affects approximately 1.4 million patients in the United States and 2.2 million people in Europe [1]. Those with IBD have a high-risk of developing colorectal cancer or toxic mega colon in the clinic [2-4] and IBD severity, such as pan-colitis and those with a disease course longer than 10 years have a higher risk in developing colorectal cancer [5,6]. To date, the precise etiology of IBD has not been elucidated, but it is now widely accepted that an inadequate activation of CD4+ T helper 1, Th 2 and Th 17 immune cells cause an imbalance between pro-inflammatory and anti-inflammatory cytokines, which plays a crucial role in IBD pathogenesis [7,8].

Specifically, IL-10 plays a pivotal role in the mucosal immune system by inhibition of pro-inflammatory cytokine synthesis and antigen presentation and in turn alleviates intestinal inflammation [9-11]. *IL-10* knockout mice spontaneously developed chronic enterocolitis resembling human CD [12]. Local delivery of plasmid carrying IL-10 cDNA ameliorated intestinal inflammation in a chemical acute colitis animal model [13-15]. Moreover, IL-4, as an anti-inflammatory cytokine, is less well elucidated in IBD. It possesses immunoregulatory and immunosuppressive effects in the gut through mediating the differentiation of naive T cells to Th2 cell and inducing Th2-type CD4+ T cells to shift towards a Th1 response [16-19]. Levels of IL-4 mRNA were shown to be decreased in IBD [20]. The efficacy of IL-4 treatment in murine IBD models is contentious [21].

In this study, we investigated the effects of IL-4 or/and IL-10 gene therapy on TNBS-induced murine colitis. We injected intraperitoneally plasmids carrying IL-4 or/and IL-10 cDNA into mice with TNBS-induced murine colitis and tested the effects of transgenic expression on TNBS-induced murine colitis. We also measured the colon tissue levels of IFN-γ, TNF-α and IL-6. Taken together, evaluation of the protective effect of IL-4 on IBD might shed light on alternative gene therapy of IBD.

## Methods

### Plasmids

The recombinant plasmids, pcDNA3.0-mIL-4 and pcDNA3.0-mIL-10, were constructed in our laboratory. Briefly, murine IL-4 or murine IL-10 gene was obtained by RT-PCR, was then integrated into the respective eukaryotic expression plasmid pcDNA3.0, and the recombinant plasmid was finally confirmed by DNA sequencing.

### Mice and TNBS-induced colitis

In this study, animal protocols were approved by the Institutional Animal Care and Use Committees of Southern Medical University. Specifically, male BALB/c mice (6–8 weeks of age with a body weight of 18–22 g) were group-housed at our animal care facility under a controlled temperature (25°C) and light–dark cycle (12:12 h). The animals were allowed unrestricted access to food and tap water. To induce the murine colitis, we followed the procedures described by Wirtz S *et al.*[22]. Briefly, the mice were first pre-sensitized by using 1.5 mg TNBS (Sigma-Aldrich, St. Louis, MO) applied to the skin. After 7 days, the mice were lightly anesthetized by intraperitoneal injection of pentobarbital sodium salt and then rectally administered 2.5 mg of TNBS dissolved in 100 μl of 50% (v/v) ethanol solution using a 1.5 mm polyethylene catheter. Mice were then kept in a vertical position for 1 min. Control mice were administered 50% ethanol in a similar manner (n = 10 mice in each group).

### Gene transfection of TNBS-treated mice

To assess the effect of pcDNA3.0-IL-4 or pcDNA3.0-IL-10 on TNBS-induced colitis, we intraperitoneally injected these plasmids into mice mixed with liposome 24 h after TNBS injection (Table 1) according to previous studies [23-30]. In brief, 100 μg plasmids were mixed with 30 μl LipofectAMINE 2000 and then incubated for 20 min at the room temperature, and injected into mice. After that, daily weight, stool consistency, rectal bleeding and animal behavior were recorded for up to 7 days. Mice were sacrificed on day 7 and the murine colon was removed for analyses of histology, INF-γ, TNF-α and IL-6 levels.

**Table 1:** Animal experiments

**Group**	**Number of mice**	**TNBS**	**Treatment**
1	10	No	30 μl LipofectAMINE
2	10	TNBS	30 μl LipofectAMINE
3	10	TNBS	100 μg pcDNA3.0 + 30 μl LipofectAMINE
4	10	TNBS	100 μg pcDNA3.0-mIL-4 + 30 μl LipofectAMINE
5	10	TNBS	100 μg pcDNA3.0-mIL-10 + 30 μl LipofectAMINE
6	10	TNBS	50 μg pcDNA3.0-mIL-4 + 50 μg pcDNA3.0-mIL-10 + 30 μl LipofectAMINE

### Evaluation of TNBS-treated mice

Body weight, stool consistency and gross bleeding were monitored daily as described by Ganta *et al.*[31]. Disease activity index (DAI) was determined by combined scores of body weight loss, stool consistency and gross bleeding. The scores were defined as follows: change in body weight (0: none, 1: 1-5%, 2: 5-10%, 3: 10-15%, 4: >15%), stool consistency (0: normal, 2: loose stool, 4: diarrhea) and stool blood (0: negative, 2: positive, 4: gross bleeding). Body weight loss was calculated as the percent difference between the original body weight (day 0) and the body weight on any given day.

### Histological analysis of colitis

At the end of experimenting, the distal colon was removed from the mice and fixed in 4% paraformaldehyde, embedded in paraffin, sectioned at 5 μm, and stained with hematoxylin and eosin (HE). Histology of murine colon tissues was independently evaluated by two experienced pathologists in a blinded fashion. Histological changes were graded from 0 to 4: 0, no sign of inflammation; 1, very low level of leukocyte infiltration; 2, low level of leukocyte infiltration; 3, high level of leukocyte infiltration, high vascular density and thickening of the colon wall; and 4, transmural infiltration, loss of goblet cells, high vascular density and thickening of the colon wall according to a previous study [32].

### Quantitative-RT-PCR

Total cellular RNA was isolated from 50 mg colon tissue using a Trizol Reagent (Invitrogen) according to the manufacturer’s instruction and then reverse-transcribed into cDNA using the M-MLV reverse transcriptase (Invitrogen) following the manufacturer’s instructions.

For qPCR, 2 μl of reverse transcription mixture was subjected to PCR amplification of IL-4, IL-10, TNF-α, IL-6 and β-actin mRNA using SYBR Green (TaKaRa, Tokyo, Japan) in the LightCyler 480 instrument (Roche Diagnostics Corporation, Indiana, USA) for 40 cycles. PCR primers were as follows: IL-4, 5′-GGTCTCAACCCCCAGCTAGT and 5′-GCCGATGATCTCTCTCAAGTGAT; IL-10, 5′-GCTCTTACTGACTGGCATGAG-3′ and 5′-CGCAGCTCTAGGAGCATGTG-3′; TNF-α, 5′-CAGACCCTCACACTCAGATCATCTT-3′ and 5′-CCTCCACTTGGTGGTTTGCT-3′; IL-6, 5′-AGAGGATACCACTCCCAACAGAC-3′ and 5′-AGTGCATCATCGTTGTTCATACAA-3′; and β-actin, 5′-GATGACCCAGATCATGTTT-3′ and 5′-ACGACCAGAGGCATACAG-3′. The PCR amplification was in duplicate. The differences in target gene expression were expressed relatively to the housekeeping gene as 2ΔΔCT, where ΔΔCT = average of ΔCT control – ΔCT treated.

### ELISA measurement of IFN-γ levels

To detect IFN-γ levels in murine colon tissues, we resected approximately 30 mg colon tissues from control and experimental mice which we homogenized in 150 μl NP-40 lysis buffer and centrifuged at 14, 000 g for 30 min. The supernatants were collected and assayed for IFN-γ protein levels by using an ELISA kit (Raybio, Bruges, Belgium) according to the manufacturer’s instructions.

### Statistical analyses

The data were represented as mean ± standard deviation (SD) and analyzed using one-way ANOVA. Repeated measure ANOVA test was used to analyze the differences in the DAI score and body weight changes between the groups. The nonparametric data (such as HE scores or levels of IFN-γ were analyzed by the Kruskal-Wallis test. All statistical analyses were performed by using SPSS 13.0 software (SPSS, Chicago, IL). P < 0.05 was considered statistically significant.

## Results

### Detection of IL-4 and IL-10 transgene expression in vivo

**Figure 1:**
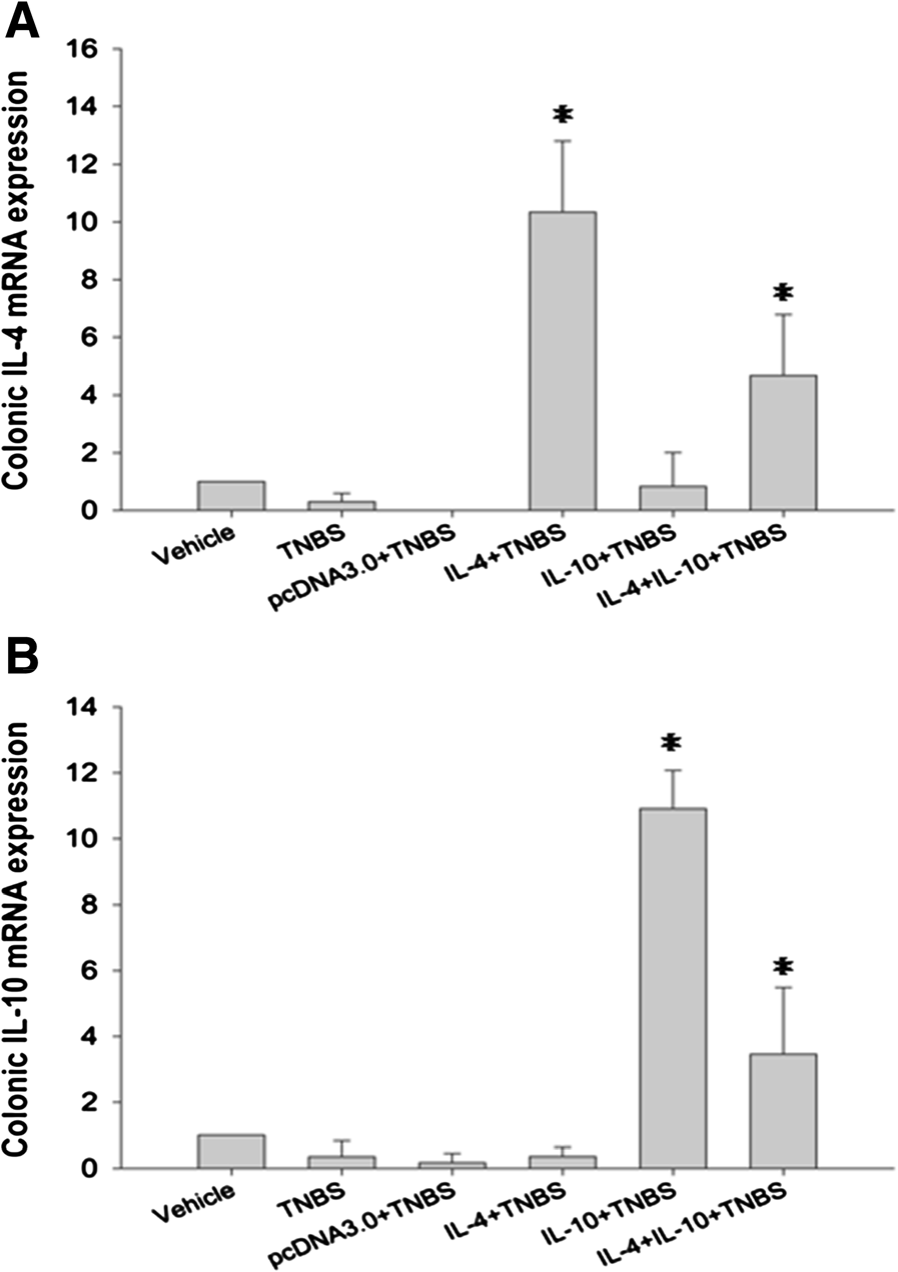
**Quantitative RT-PCR detection of IL-4 and IL-10 expression *in vivo*.** Colon tissue samples were removed from TNBS-treated murine colitis on day 7 and subjected to RNA isolation and qRT-PCR. **A**: The expression of IL-4 mRNA in vivo. **B**: The level of IL-10 mRNA in vivo. The transgenic expression of IL-4 or IL-10 was successfully induced. All data are expressed as mean ± s.d.

To assess the effects of IL-4 or/and IL-10 expression on treatment of TNBS-induced murine colitis, we intraperitoneally injected these plasmids into mice and then detected these transgenic expressions in murine colon tissues using qRT-PCR (Figure 1). In brief, the level of IL-4 mRNA in the mice injected with IL-4 plasmid and in the combination group was significantly higher than that of pcDNA3.0 injection and TNBS-induced mice. Similarly, the level of IL-10 mRNA was also significantly higher in the mice injected with IL-10 plasmid and the combination group than pcDNA3.0 injection and TNBS-induced mice. These data demonstrated that intraperitoneal injection of these plasmids was able to induce the transgenic expression in murine colon tissues.

### Effects of IL-4 and IL-10 on treatment of TNBS-induced microscopic injury of colon tissues

**Figure 2:**
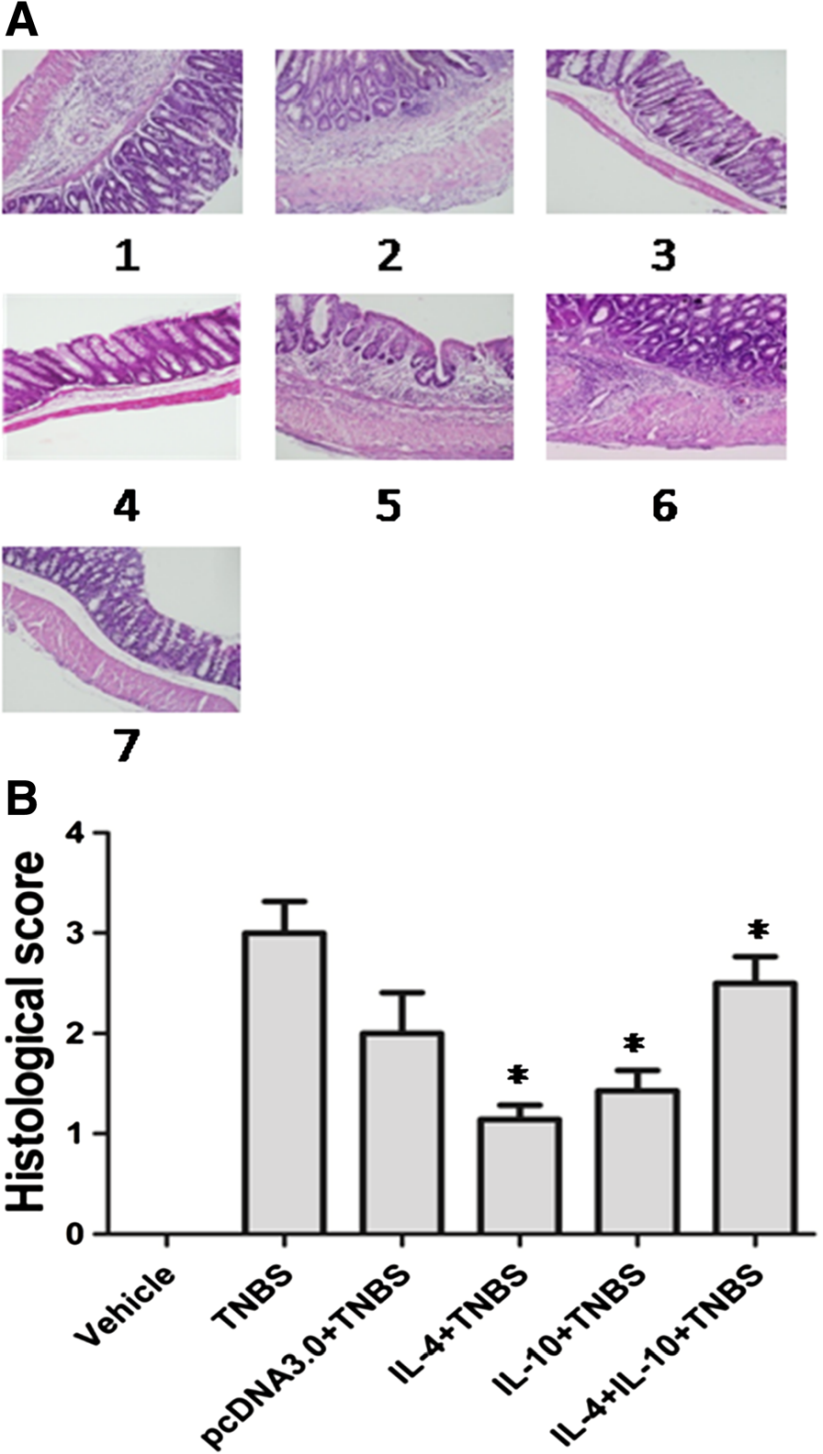
**Effects of IL-4 or IL-10 transfection on treatment of TNBS-induced murine colitis.** IL-4 or IL-10 treatment attenuated colonic inflammation and reduced histological colitis scores in TNBS-induced murine colitis. In contrast, their combination has minimal effect on preventing colitis development. **A**: Hematoxylin and eosin staining of the colon. Group 1, treated with TNBS only; Group 2, treated with pcDNA3.0; Group 3, treated with IL-4; Group 4, treated with IL-10; Group 5, treated with combination. Magnification, ×200. Scale bar = 100 μm. **B**: Histologic scores of the colon tissues. Each tissue section was evaluated for an inflammatory score from 0 to 4 and the data are expressed as median (*p < 0.05 compared to the TNBS-only group using Kruskal Wallis test).

In the TNBS-induced colitis model, we found that TNBS induced the colon tissues to severe inflammation, *e.g.* thickening of the colon wall and a high level of leukocyte infiltration (Figure 2A). In contrast, IL-4 and IL-10 transgenic expression prevented colitis histology, *i.e.*, histology of IL-4, IL-10 or their combination-treated colon tissues appeared almost normal, with a low level of infiltrating leukocytes (Figure 2A). Figure 2B summarized the median histological score and showed that TNBS-treated mice had prominently higher histological scores than that of all other groups. Mice treated with IL-4, IL-10 or their combination had significantly lower histological scores than that of TNBS only-treated mice. However, there was no obvious difference in the histological scores among mice administrated with combination pcDNA3.0 plus TNBS. These data showed that a single injection of IL-4 or IL-10 was sufficient to ameliorate colitis.

### Effects of IL-4 or IL-10 on treatment of TNBS-induced murine body weight and disease severity

**Figure 3:**
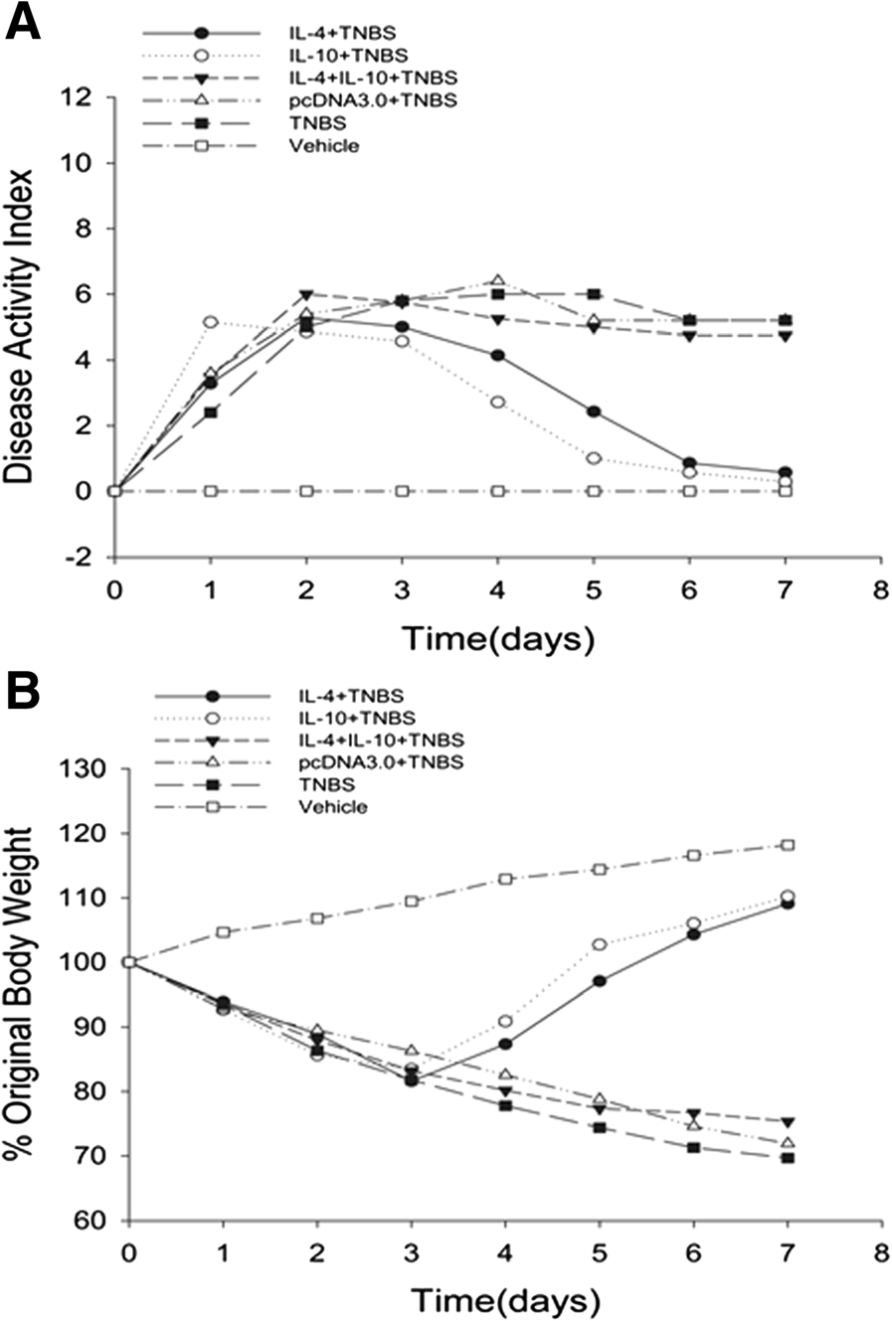
**Effects of IL-4 or IL-10 on treatment of TNBS-induced mouse body weight and disease severity. A**: Disease activity index. **B**: Body weight changes. All data are expressed as mean ± s.d.

The changed body weight of each mouse was measured every day by comparing the current weight with the weight on day 0 and the disease activity was also monitored (Figure 3A). Compared with mice treated with IL-4 or IL-10 after TNBS induction, the body weight of mice treated with the combination treatment decreased significantly. Compared with the control mice, the body weight of mice administered with IL-4 or IL-10 had significant recovery in day 3. Figure 3B showed the data of disease activity index, *i.e.*, the disease activity score of IL-4 or IL-10 mice nearly returned back to normal on day 7, whereas the combination group maintained a high level, similar to TNBS only-treated mice. These data indicate that administration of IL-4 or IL-10 significantly attenuated body weight loss and disease severity after TNBS-induced colitis, whereas the combination treatment had no significant effect on this process.

### Effects of IL-4 or IL-10 on suppression of TNBS-induced IFN-γ levels in TNBS-induced murine colitis

**Figure 4:**
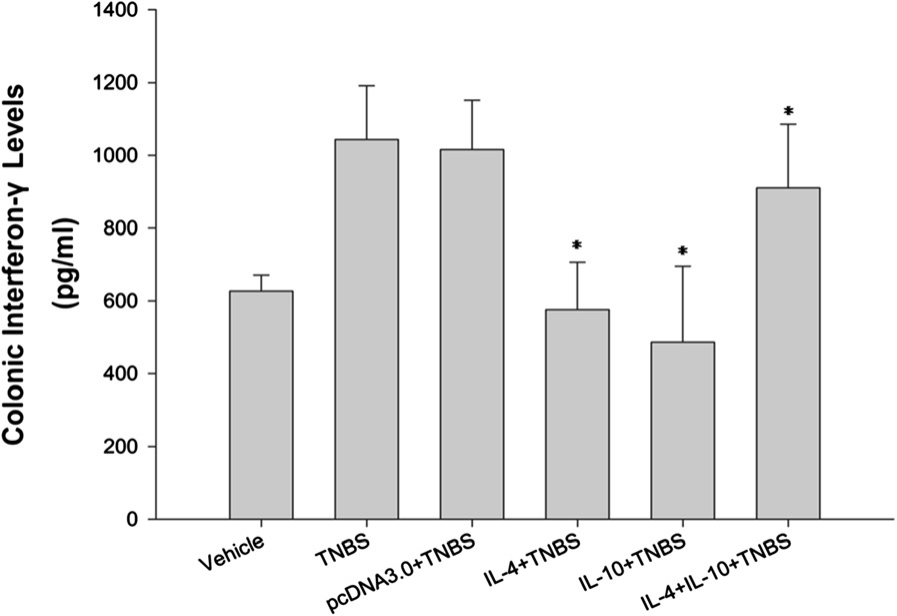
**Effects of IL-4 or IL-10 on suppression of TNBS-induced IFN-γ levels in TNBS-induced murine colitis.** Colonic IFN-γ levels were measured by using ELISA and the data are expressed as median (*p < 0.05 compared to the TNBS-only group using Kruskal Wallis test).

The acute colitis induced by TNBS administration is associated with the release of pro-inflammatory cytokines. In order to determine the effect of IL-4 or IL-10 therapy on TNBS-induced murine colitis, colonic levels of IFN-γ protein were assessed using ELISA on all mice on day 7 and the results showed that TNBS-treated mice had elevated levels of IFN-γ (1043.804 pg/ml), whereas IL-4 or IL-10 therapy significantly reduced levels of IFN-γ protein (593.116 pg/ml and 421.204 pg/ml, respectively). In contrast, the combination therapy has lower effects (898.469 pg/ml vs. 593.116 pg/ml or 421.204 pg/ml, respectively) (Figure 4).

### Effects of IL-4 or IL-10 on suppression of TNBS-induced TNF-α and IL-6 expression in TNBS-induced murine colitis

**Figure 5:**
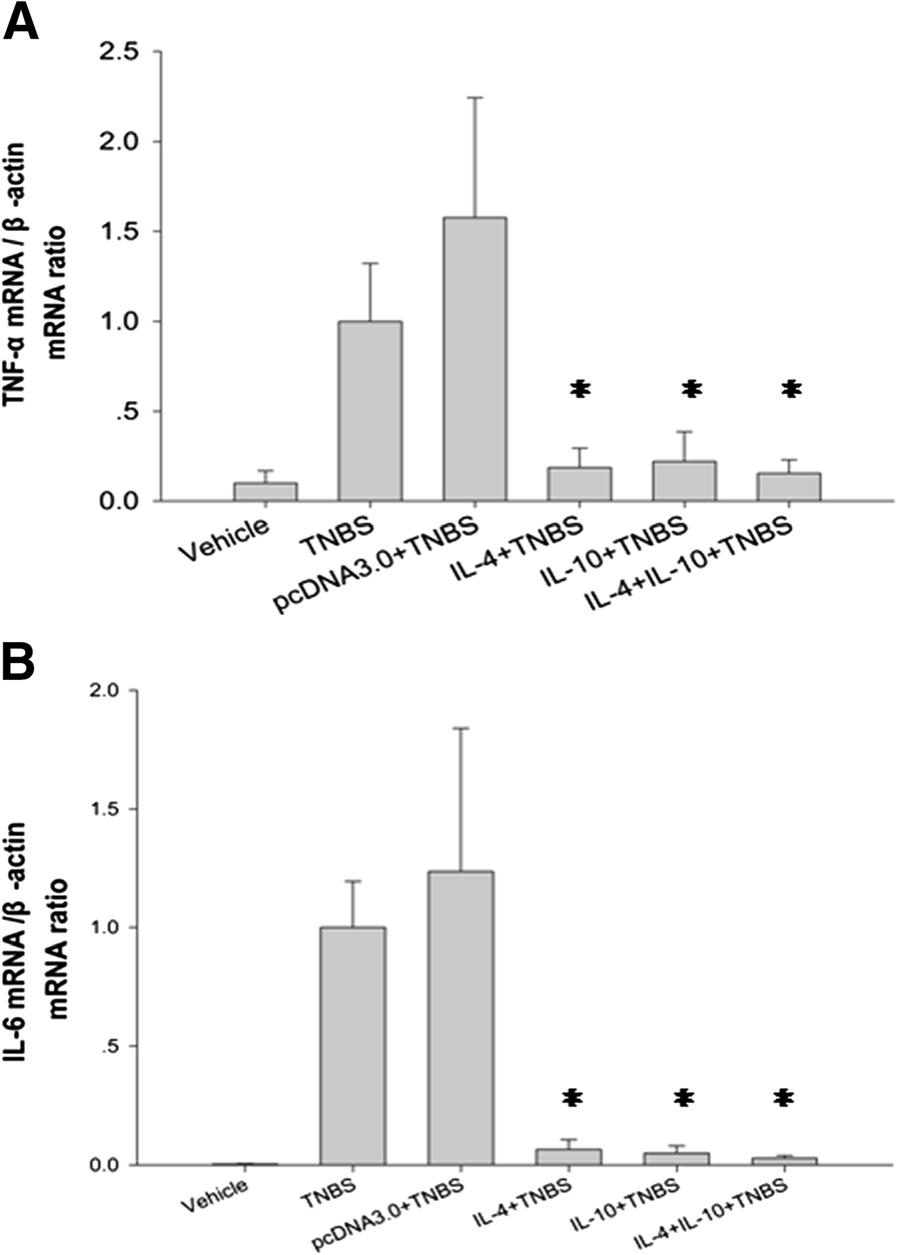
**Effects of IL-4 or IL-10 on suppression of TNBS-induced TNF-α and IL-6 expression in TNBS-induced murine colitis.** Mice with TNBS-induced colitis showed a higher level of pro-inflammatory cytokines (TNF-α and IL-6) than that of the mice treated with IL-4, IL-10 and their combination. **A**: The colonic TNF-α expression was determined by quantitative real-time RT-PCR. **B**: The expression of colonic IL-6 in mice. The data are expressed as mean ± s.d. (*p < 0.05 vs. the Control using one-way ANOVA with Dunnett's T3 test).

To further confirm the therapeutic effect of IL-4 and IL-10 gene therapy, we performed qRT-PCR analysis of their expression in TNBS-induced murine colitis tissues. We found that TNBS treatment alone induced expression of TNF-α and IL-6 mRNA in the colon tissues, whereas administration of IL-4, IL-10 or their combination significantly down-regulated expression of mucosal TNF-α and IL-6 mRNA in the colon tissues (Figure 5).

## Discussion

A number of animal models of intestinal inflammation, indispensable for our understanding of the pathogenesis of IBD and testing of novel therapeutics, have been established by using chemical induction, immune cell transfer or genetic manipulations [33]. A frequently used murine model is based on the intrarectal administration of TNBS and produced human-like CD and is characterized by Th1-mediated inflammation [34]. Our current data confirmed this TNBS-induced colitis model with elevated Th1-like cytokines. We also showed that IL-4 or IL-10 gene therapy had a markedly lower disease severity than that of the TNBS alone-treated group. The body weight of the treated mice was drastically recovered from day 4 to day 7 and the levels of IFN-γ protein, TNF-α and IL-6 mRNA were remarkably down-regulated after IL-4 or IL-10 gene therapy compared to TNBS alone-treated mice. It is very interesting for us to find that the combination of both cytokines had less effects that that of each individual one.

Our current study is consistent with previous reports on the role of IL-10 in intestinal inflammation [13-15]. Moreover, Barbara *et al*. showed that adenovirus carrying IL-10 suppressed experimental colitis in rat [35]. Lindsay and his colleagues also showed that local IL-10 gene therapy using an adenoviral vector reversed colitis in IL-10−/− mice after intravenous administration [36]. Moreover, our current data also confirmed the effects of IL-4 on experiment colitis. This finding is consistent with the data shown by Hogabaom *et al.*[37]. In fact, despite the accumulated knowledge from experimental IBD models, the biology of IL-4 *in vivo* remains controversial. For example, IL-4 was shown to be either beneficial or detrimental in different experimental settings. Hogabaom *et al.* demonstrated that IL-4 delivered by adenovirus-5 was therapeutic for acute TNBS-induced rat colitis, which was associated with an inhibition of inducible nitric oxide expression and a reduction in nitric oxide synthesis. However, the acute DSS-induced colitis was exacerbated in IL4+/+mice, and not in IL4−/− mice [21]. Accordingly, Madeline *et al.* revealed that IL-4 indirectly promoted Th1-type inflammation in the CD4^+^CD45^RBhigh^ T cell transfer model of colitis, while co-treatment with IL-10 blocked the development of colitis [38]. Possible explanations for the discrepancy may be because of variances in animal models used (induction of disease, chemical versus T cells or animals, rats versus mice), method of IL-4 administration (adenovirus versus liposome mediated plasmid delivery, time of cytokine addition (pre versus post). In addition, the site of injection, administrated dosage, and milieu of cytokines may also be factors in affecting biological activity of exogenous IL-4 in these IBD models.

In our current study, the mechanisms responsible for IL-4 or IL-10 anti-colitis could be due to control of pro-inflammatory cytokine production and immune cells accumulation in gut tissues. TNF-α and IFN-γ are master cytokines in the pathogenesis of IBD. TNF-α can increase IL-1β, IL-6 and IL-33 production as well as modulate ST2 expression in epithelial cells [39,40]. The serum levels of TNF-α and sIL-6R were significantly increased in patients with active UC and CD and correlate with the clinical activity of UC and CD [41,42]. IL-6 signaling *via* signal transducer and activator of transcription-3 (STAT3) plays an important role in UC pathogenesis [43]. On the other hand, recent studies also verified an important role for IL-6 in the suppression of Treg function and in the development of pathogenic Th17 cells, which are also involved in IBD models [7,44].

There may be three potential explanations why the combination treatment has less effect as a therapeutic strategy, *i.e.*, i) The administered dose of the combination (half of each single dose) may not reach the effective dose to elicit a protective response; ii) Their combination may not be at their optimal concentrations for producing clinical effects; and iii) Their immune-stimulatory effects counterbalance their immune-suppressive properties. Co-treatment with IL-10, IL-4 may exert pro-inflammatory actions through synergizing with TNF-α to induce adherence of eosinophil and basophil to endothelium, and synergizing with IFN-γ to increase secretory component expression by epithelial cells [45-47]. Thus, this study is just a proof-of-principle and we did not determine how IL-4 or IL-10 exerts its effect in the current animal model and which cell types are responsible for mediating these complex cell/cytokine interactions. Future work will utilize these plasmids to test the effects of IL-4 and IL-10 in other chronic murine inflammatory bowel disease models. We will also explore the biological significance of these cytokines in human IBD.

## Conclusions

In the current study, we investigated the effects of IL-4 or/and IL-10 gene therapy against TNBS-induced murine colitis and found that the liposome-mediated combination gene therapy had a significant efficacy in treatment of TNBS-induced murine colitis. Specifically, treatment of mice with IL-4 or IL-10 resulted in a marked improvement in the histological appearance of the distal colon and suppression of Th1-cell type cytokines, such as IFN-γ, TNF-α and IL-6 in TNBS-induced murine colitis. However, treatment with combination cytokines did not yield a similar effect as individual treatment. The intraperitoneal injection of cytokine plasmid plus liposome was well tolerated in the mice. Liposome did not induce an acute phase response nor exacerbate intestinal inflammation in control mice. Thus, the current gene delivery method may be useful as a potential treatment modality. Future study will determine their long-term effects in animal experiments before applying this method to human study.

## Competing interests

The authors declare that they have no competing interests.

## Authors’ contributions

YB and JX: substantial contributions to research conception and study design; JX: acquisition of data, drafting the manuscript; Y-HL and L-HB: analysis and interpretation of data; J-D W: critically read and revised this manuscript with important intellectual content; YB and SL: Study design and discussion of revision of this manuscript. All authors read and approved the final manuscript.

## Pre-publication history

The pre-publication history for this paper can be accessed here:

http://www.biomedcentral.com/1471-230X/13/165/prepub
